# Cost-Effectiveness of a Diabetes Pay-For-Performance Program in Diabetes Patients with Multiple Chronic Conditions

**DOI:** 10.1371/journal.pone.0133163

**Published:** 2015-07-14

**Authors:** Hui-Min Hsieh, Song-Mao Gu, Shyi-Jang Shin, Hao-Yun Kao, Yi-Chieh Lin, Herng-Chia Chiu

**Affiliations:** 1 Department of Public Health, Kaohsiung Medical University, Kaohsiung, Taiwan; 2 Division of HIV/AIDS and TB, Centers for Disease Control, Taipei, Taiwan; 3 Center for Lipid and Glycomedicine Research and College of Medicine, Kaohsiung Medical University, Kaohsiung, Taiwan; 4 Division of Endocrinology and Metabolism, Kaohsiung Medical University Hospital, Kaohsiung, Taiwan; 5 Department of Healthcare Administration and Medical Informatics, Kaohsiung Medical University, Kaohsiung, Taiwan; 6 Department of Business Administration, National Sun Yat-Sen University, Kaohsiung, Taiwan; Mathematical Institute, HUNGARY

## Abstract

Pay for performance (P4P) has been used as a strategy to improve quality for patients with chronic illness. Little was known whether care provided to individuals with multiple chronic conditions in a P4P program were cost-effective. This study investigated cost effectiveness of a diabetes P4P program for caring patients with diabetes alone (DM alone) and diabetes with comorbid hypertension and hyperlipidemia (DMHH) from a single payer perspective in Taiwan. Analyzing data using population-based longitudinal databases, we compared costs and effectiveness between P4P and non-P4P diabetes patient groups in two cohorts. Propensity score matching (PSM) was used to match comparable control groups for intervention groups. Outcomes included life-years, quality-adjusted life-years (QALYs), program intervention costs, cost-savings and incremental cost-effectiveness ratios (ICERs). QALYs for P4P patients and non-P4P patients were 2.80 and 2.71 for the DM alone cohort and 2.74 and 2.66 for the DMHH patient cohort. The average incremental intervention costs per QALYs was TWD$167,251 in the DM alone cohort and TWD$145,474 in the DMHH cohort. The average incremental all-cause medical costs saved by the P4P program per QALYs were TWD$434,815 in DM alone cohort and TWD$506,199 in the DMHH cohort. The findings indicated that the P4P program for both cohorts were cost-effective and the resulting return on investment (ROI) was 2.60:1 in the DM alone cohort and 3.48:1 in the DMHH cohort. We conclude that the diabetes P4P program in both cohorts enabled the long-term cost-effective use of resources and cost-savings, especially for patients with multiple comorbid conditions.

## Introduction

The increase in patients with multiple chronic conditions (MCCs) poses a great challenge to many countries’ healthcare systems, especially those with aging populations [1, 2]. Diabetes has a global prevalence of 8.3%, and more than 90% of people with diabetes have one or more comorbid chronic conditions [3–5]. Hypertension and hyperlipidemia are two often uncontrolled modifiable cardiovascular disease (CVD) risk factors in patients with type 2 diabetes [6]. Based on National Health Interview Surveys, the U.S. found a 9% and 15% increase in diabetes and hypertension between 1999 and 2009 [7]. Similarly, Taiwan has seen a more than a two-fold increase in the number of cases of diabetes with comorbid hypertension and hyperlipidemia from 10.47% in 2000 to 25.65% in 2009 [8]. Such patients have a lower quality of life, higher utilization of health care services and related costs, and significantly higher risk of CVD-related morbidity and mortality [9–11]. Therefore, to provide patient-centered care for those multi-morbid diabetic patients became important priority in many countries [2].

Pay for performance (P4P) has been embraced by many developed nations as a strategy to improve healthcare delivery and quality for patients with chronic illness. For examples, the United Kingdom National healthcare system and Australia P4P programs pay extra bonuses for reward improvement for providing good quality of care for diabetes patients [12, 13]. A P4P can be considered cost-effective when quality is improved at equal or lower costs. However, P4P quality of care schemes are normally based on single-disease clinical practice guidelines [1, 4, 14], little were known whether care provided to individuals with multiple comorbid conditions in such a P4P program receive equal or better cost-effective compared with individuals with single disease. Patients with multiple chronic conditions often require more effort and time for health providers to achieve targeted outcomes under a P4P program. Nonetheless, frequent and regular P4P outpatient follow-up visits may decrease the risks of any diabetes-related end point (e.g., microvascular disease, myocardial infarction or death) for those people in need [15], which would in turn reduce health utilization and health expenses related to emergency visits or hospitalizations.

Existing studies on the cost-effectiveness (CE) of P4P are scarce and inconclusive [16–18], not to mention an evaluation on cost-effectiveness of P4P on patients with multiple comorbid conditions particularly. Most have focused on the quality improvements of P4P programs, but have neglected their cost and cost-effectiveness [16, 17, 19–21]. This knowledge gap has been noted by a number of reviews [16, 17, 19–21]. Few studies have attempted to estimate cost-effectiveness using full economic evaluations (e.g., quality adjusted life-years, QALYs) [16, 19]. For example, two studies conducted comprehensive cost-effectiveness or cost-utility analysis of a P4P program among hospitals participating in P4P and those not participating in the program in England and in the United States [19, 22]. Tan et al. (2014) recently also conducted a cost-utility analysis at the patient level from a single payer perspective to investigate whether a diabetes P4P program was cost-effective during 2004–2005 in Taiwan [23]. Hsieh et al. (2015) compared the cost-effectiveness of changes in P4P incentive designs among overall diabetes patients for a period 2002 to 2006 and a 2007 to 2011 period in Taiwan [24]. These two studies, however, only focus on single-disease perspective. To date, no study has examined to what extent of cost-effectiveness of a P4P program on caring patients with multiple chronic conditions.

The purpose of this study is to investigate whether a diabetes P4P program allowed for a cost-effective use of resources and to what extent their cost effectiveness differed for caring patients with Diabetes alone and multiple comorbid conditions (hypertension and hyperlipidemia) from a single payer perspective in Taiwan. Taiwan National Health Insurance (NHI) implemented a diabetes P4P program aimed at improving quality in health care for diabetes patients. Physicians who specialized in metabolic disorders or endocrinology or those who had participated in a training program for diabetes shared care were eligible to participate in the program [25]. They and their medical care staff members at the various hospitals and clinics were expected to work as a coordinated physician-led multi-disciplinary team adhering to clinical guidelines established for the care of diabetes patients [26]. We conducted longitudinal retrospective observational cohort study using population-based longitudinal national health insurance claims data for a 2007 to 2012 period. Our primary objective was to examine the cost-effectiveness of P4P in patient cohorts with Diabetes alone and multiple comorbid conditions (hypertension and hyperlipidemia), and to investigate the incremental gains in cost-effectiveness between two groups. We hypothesized that the long-term cost-effectiveness may be greater in the patients with multiple comorbid conditions under the P4P program scheme. The results of such a study could help in future P4P program development on care for multiple chronic conditions.

## Methods

### Study Design and Data Sources

We conducted a longitudinal observational cohort study design using three nationwide population-based databases in Taiwan for a 2007 to 2012 period. One database was a nationwide diabetes P4P database from which we could precisely identify whether patients were enrolled in the P4P program. Another was the NHI administrative claims database from which we could obtain information on patient comorbidities and health provider characteristics. The other was a database containing death registry data, which provides accurate death date information. Due to the legal and privacy protection policy in the grant contract according to the NHI administration (NHIA) policy, data was restricted and not available to public. Data was only available for researchers who have grant contracts with the NHIA to access anonymized and de-identified patient records. This study received ethical approval from the Institutional Review Board (IRB) (KMUH-IRB-20130019) in Kaohsiung Medical University Hospital in Taiwan.

### Study Population

Using nationwide NHI claims data, we included type 2 diabetes patients if he or she had primarily diabetes diagnosis (ICD-9-CM codes with 250.xx or A-code 181, excluding 250.x1 or 250.x3) in at least two outpatient visits or at least one inpatient hospitalization for each year during 2007 and 2008. Using the P4P database, we identified newly enrolled P4P patients as study P4P cohorts during the patient identification period and defined the date for each P4P patient as the date that they were first enrolled in the P4P program as index date. Patients younger than 18 years old at index date were excluded. We then identified non-P4P diabetes patients as comparison groups if those patients were not found to be enrolled in the P4P program during the above-stated time period. The **S1 Fig** provides more information about study inclusion and exclusion criteria.

To address the issue of patient having multiple outpatient visits to different healthcare providers, we applied the plurality provider algorithm for assigning a non-P4P patient to the most frequently seen physician, defined as one who billed for the greatest number of care visits during identification period, as has been used in previous studies [25, 27, 28]. We directly assigned P4P patients to the physician who enrolled them into the P4P program as the most frequently seen physician. In total, there were 11,894 physicians treating 76,901 of P4P patients and 826,612 of non-P4P patients. About 9.30% of diabetes populations were newly enrolled in the P4P program.

We further identified patients with hypertension and hyperlipidemia from those diabetes patients from NHI administrative claims within one year prior to the enrollment date for each patient. We first classified patients with hypertension if they have at least two outpatient visit or inpatient hospitalization with ICD-9-CM diagnosis codes 401.xx; or have been prescribed at least one anti-hypertensive medication on the basis of the Anatomical Therapeutic Chemical Classification System (ATC codes) with anti-hypertensive, diuretics, beta blocker, calcium channel blockers and agents acting on the renin-angiotensin system. We then also classified patients with hyperlipidemia if they have at least two outpatient visits or inpatient hospitalization with ICD-9-CM diagnosis codes 272.xx; or have been prescribed at least one anti-hypertensive medication on the basis of ATC code with lipid modifying agents. For more details about ATC codes for the antihypertensive and anti-lipidemic drugs, please see the **S1 Table**. After excluding numbers of diabetes patients with only hypertension and with only hyperlipidemia, a total 14,267 of P4P and 220,383 of non-P4P patients with diabetes alone (DM alone) and 29,566 of P4P and 202,944 of non-P4P diabetes with comorbid hypertension and hyperlipidemia (DMHH) were included in the final analysis.

To avoid potential confounding by selection bias and confounding factors, we used propensity score matching approach (PSM) to determine comparison groups. Using a logistic regression model, we created propensity scores that predicted the probability of patients’ enrollment in the P4P program in DM alone and the DMHH cohorts separately. The covariates included patients’ demographic characteristics (age and gender), a diabetes complication severity index (DCSI) [29], a chronic illness with complexity (CIC) index [30, 31]. The DCSI takes into consideration seven categories of complications (identified by ICD-9-CM codes): cardiovascular complications, nephropathy, retinopathy, peripheral vascular disease, stroke, neuropathy, and metabolic disorders. DCSI has a total score of 13 points, the higher score, the more severe the disease state. The CIC index was used to adjust for comorbidity of patients with multiple chronic diseases. This index covers non-diabetes physical illness complexity (including cancers, gastrointestinal, musculoskeletal, and pulmonary diseases), diabetes-related complexity, and mental illness/substance abuse complexity. Diabetes-related complexity of CIC was not included to avoid duplication comorbidity-related values captured by the DCSI index [25]. Following previous studies [25, 27], both DCSI and CIC measures were categorized into three categories (0, 1, and > = 2). In addition, because the P4P program required health staff worked as a team to care patients and cost structures may differ in different level of health institutions, health care provider characteristics were also included, such as accreditation level (medical center, regional hospital, local hospital or clinic), ownership type (public, not-for-profit, or for-profit), and geographic location (Taipei, northern, central, southern, Kao-Ping, or eastern area), to capture the resources and capacities for individual health care institutions.

The PSM caliper matching method with 1 to 1 match was used to match intervention group members with comparison group based on propensity score [32, 33]. Given that non-P4P patients lacked specific enrollment index dates, their index date was assigned based the index date of their matched counterpart in their corresponding P4P group. To compare between groups, we followed each P4P and non-P4P patient for four years from the index date. Any patient was censored if he or she dropped out of the insurance program or had died.

### Cost Measures

We analyzed costs from a single payer perspective. Two distinct types of direct medical costs were measured. The first was the P4P program intervention cost, which was measured using diabetes-related outpatient costs (“DM-OPD costs”). Given that the P4P programs paid physicians quality bonuses for providing essential examinations/tests outlined by the program in addition to regular DM-OPD diabetes care and thus P4P patients may visit physician office more frequently than non-P4P patients, the difference of DM-OPD treatment costs between P4P and non-P4P patients would be reflected in the program intervention costs. Second, we measured cost-savings from P4P program using two parameters. One was the potential cost-savings resulting from reduced costs in diabetes-related emergency visits and hospital admissions, which were measured as diabetes-related medical costs (“DM-ED/INP costs”), and the other was all-cause medical costs with the exclusion of the DM-OPD costs. It is assumed that improved quality of care through regular outpatient follow-up visits would decrease the risks of any diabetes-related end point (e.g., microvascular disease, myocardial infarction or death)[15], which would in turn reduce health utilization and health expenses related to emergency visits or hospitalizations. Cost data were extracted from the NHI claims and adjusted to 2007 price based on the NHI global budgeting annual negotiation rate (approximately 3% discount rate). Costs are presented in Taiwan Dollar (TWD). The exchange rate between TWD and USD dollars is about 1:30 in this study.

### Effectiveness Measures

We used patients’ life-years saved (LYs) and quality-adjusted life years saved (QALYs) as effectiveness measures because intensive diabetes care may decrease risks of complications or death and thus increase life years [15]. Life-years were measured from the index date till death or the date of last follow-up within four years in the censored data. Mean general utility weighted was calculated as proxy measures to capture health-related utility for P4P and non-P4P diabetes patients in this study. Specifically, we used survey data from the generic Short-Form (SF) 12 survey instrument, which is a multidimensional generic measure of health-related quality of life for chronic care [34, 35], to estimate health-related quality of life (HRQoL). Following previous published preference-based algorithms, the SF-12 scores were converted to utility weights, ranging from 0 to 1 [34–36]. The higher utility weight, the better the health-related quality of life. This data were derived from one of our working studies, which is a large-scale nationwide cross-sectional survey of diabetes patients with and without enrollment in Taiwan’s diabetes P4P program from February to November in 2013. As shown in the S2 Table, the total number of type 2 diabetes in the survey was 1,296 (938 P4P patients, 357 non-P4P patients). The overall utility weight for P4P and non-P4P patients in our study were 0.71(±0.01) and 0.70(±0.02). QALYs were then measured by multiplying life-years by utility weight of each patient in both P4P and non-P4P groups.

### Economic and Statistical Analytical Approach

In order to answer the study questions, we conducted a cost-utility analysis. We analyzed the costs over the 4-year period for each patient and discounted the effects over the expected patient life. We analyzed data using multiple generalized linear regressions while controlling for variations from patient characteristics and health care provider factors for DM alone and the DMHH cohorts separately. A heteroskedasticity-robust standard error adjustment was used, and patients were clustered within health care institutions to control for unequal error variances across institutions. We then calculated incremental costs and effectiveness by differences in these values for P4P and non-P4P patients using adjusted predicted estimates. In addition, we calculated the incremental cost-effectiveness ratio (ICER) as the ratio of the difference in costs between P4P and non-P4P groups and divided by difference in effectiveness for each cohort [37, 38]. All incremental measures were adjusted by the patient demographic and clinical characteristics as well as health care institutional characteristics. Bootstrapping with 100 replications with sample size equivalent to the original was used to obtain standard errors for the incremental measures [39, 40]. Each point of bootstrapped estimate of the adjusted incremental effectiveness and costs were generated and then plotted in an incremental cost-effectiveness plane [37–39]. All statistical operations were performed using SAS version 9.3 (SAS institute, Cary, NC) and Stata SE 12 version. A p-value<0.05 was considered significant.

## Results

Tables 1 and 2 summarize baseline patient and healthcare provider characteristics for the matched P4P and non-P4P patients in the DM alone and DMHH cohorts. Before matching, we included 14,267 P4P patients and 220,383 non-P4P patients in the DM alone group, and 29,566 and 202,944 in the DMHH cohort. In both cohorts, significant differences were found between the pre-matched intervention and comparison groups (p<0.001) with respect to all characteristics assessed. After PSM for 1 to 1 matching, however, the two groups were found to be similar in both cohorts.

**Table 1:** Baseline patient and healthcare provider characteristics for P4P and non-P4P patients with diabetes alone (DM alone).

	DM alone					
	Before Matching			After Matching		
Variables	P4P	Non-P4P		P4P	Non-P4P	
	Mean ± SD/ (N, %)	Mean ± SD/ (N, %)	p-value	Mean ± SD/ (N, %)	Mean ± SD/ (N, %)	p-value
N	14,267	220,383		14,267	14,267	
**Patients' Demographic Characteristics**						
Gender						
Male (N, %)	8,205 (57.51%)	127,662 (57.93%)	0.328	8,205 (57.51%)	8,200 (57.48%)	0.952
Female (N, %)	6,062 (42.49%)	92,721 (42.07%)		6,062 (42.49%)	6,067 (42.52%)	
Age Categories (N, %)						
<45	3,568 (25.01%)	45,045 (20.44%)	<0.001	3,568 (25.01%)	3,569 (25.02%)	0.999
45–54	4,603 (32.26%)	66,721 (30.28%)		4,603 (32.26%)	4,585 (32.14%)	
55–64	3,733 (26.17%)	58,929 (26.74%)		3,733 (26.17%)	3,737 (26.19%)	
65–74	1,811 (12.69%)	32,748 (14.86%)		1,811 (12.69%)	1,816 (12.73%)	
75+	552 (3.87%)	16,940 (7.69%)		552 (3.87%)	560 (3.93%)	
**Patients' clinical characteristics**						
DCSI score (mean ± SD)	0.30 (± 0.70)	0.40 (± 0.82)	<0.001	0.30 (± 0.70)	0.30 (± 0.67)	0.298
DCSI categories (N, %)						
0	11,322 (79.36%)	163,923 (74.38%)	<0.001	11,322 (79.36%)	11,348 (79.54%)	0.763
1	1,908 (13.37%)	34,230 (15.53%)		1,908 (13.37%)	1,914 (13.42%)	
> = 2	1,037 (7.27%)	22,230 (10.09%)		1,037 (7.27%)	1,005 (7.04%)	
CIC counts (mean ± SD)	0.81 (± 0.88)	0.85 (± 0.89)	<0.001	0.81 (± 0.88)	0.81 (± 0.87)	0.583
CIC categories (N, %)						
0	6,291 (44.09%)	91,985 (41.74%)	<0.001	6,291 (44.09%)	6,310 (44.23%)	0.205
1	5,078 (35.59%)	80,287 (36.43%)		5,078 (35.59%)	5,078 (35.59%)	
> = 2	2,898 (20.31%)	48,111 (21.83%)		2,898 (20.31%)	2,879 (20.18%)	
**Health care institution characteristics**						
Accreditation level (N,%)						
Medical Center	2,345 (18.82%)	31,526 (18.98%)	<0.001	2,345 (18.82%)	2,349 (18.85%)	0.723
Regional Hospital	4,529 (36.34%)	36,998 (22.27%)		4,529 (36.34%)	4,551 (36.52%)	
Local Hospital	2,282 (18.31%)	28,830 (17.35%)		2,282 (18.31%)	2,270 (18.21%)	
Clinics	3,307 (26.53%)	68,678 (41.34%)		3,307 (26.53%)	3,291 (26.41%)	
Ownership type (N, %)						
Public	2,759 (22.14%)	37,918 (22.82%)	<0.001	2,759 (22.14%)	2,762 (22.16%)	0.941
Not-for-profit	5,104 (40.95%)	43,701 (26.30%)		5,104 (40.95%)	5,091 (40.85%)	
For-profit	4,600 (36.91%)	84,519 (50.87%)		4,600 (36.91%)	4,610 (36.99%)	
Location of health care institution (N, %)						
Taipei	3,951 (31.70%)	46,441 (27.95%)	<0.001	3,951 (31.70%)	3,933 (31.56%)	0.998
Northern	1,338 (10.74%)	25,695 (15.47%)		1,338 (10.74%)	1,357 (10.89%)	
Central	3,138 (25.18%)	28,105 (16.92%)		3,138 (25.18%)	3,127 (25.09%)	
Southern	1,747 (14.02%)	29,682 (17.87%)		1,747 (14.02%)	1,755 (14.08%)	
Kao-Ping	2,668 (18.70%)	44,168 (20.04%)		2,668 (18.70%)	2,668 (18.70%)	
Eastern	5,080 (35.61%)	49,261 (22.35%)		5,080 (35.61%)	5,099 (35.74%)	

Note: DM alone = patients with diabetes alone; DCSI = diabetes complications severity index; CIC = chronic illness with complexity.

P-value here was to compare patient demographic, clinical characteristics and health institution characteristics between P4P and non-P4P patients.

**Table 2:** Baseline patient and healthcare provider characteristics for P4P and non-P4P diabetes patients with hypertension and hyperlipidemia (“DMHH”).

	DMHH					
	Before Matching			After Matching		
Variables	P4P	Non-P4P		P4P	Non-P4P	
	Mean ± SD/ (N, %)	Mean ± SD/ (N, %)	p-value	Mean ± SD/ (N, %)	Mean ± SD/ (N, %)	p-value
N	29,566	202,944		29,518	29,518	
**Patients' Demographic Characteristics**						
Gender						
Male (N, %)	13,418 (45.38%)	93,405 (46.03%)	0.039	13,405 (45.41%)	13,361 (45.26%)	0.716
Female (N, %)	16,148 (54.62%)	109,539 (53.97%)		16,113 (54.59%)	16,157 (54.74%)	
Age Categories (N, %)						
<45	2,439 (8.25%)	11,968 (5.90%)	<0.001	2,403 (8.14%)	2,429 (8.23%)	0.996
45–54	6,728 (22.76%)	37,452 (18.45%)		6,716 (22.75%)	6,716 (22.75%)	
55–64	9,308 (31.48%)	57,259 (28.21%)		9,308 (31.53%)	9,314 (31.55%)	
65–74	7,864 (26.60%)	60,518 (29.82%)		7,864 (26.64%)	7,839 (26.56%)	
75+	3,227 (10.91%)	35,747 (17.61%)		3,227 (10.93%)	3,220 (10.91%)	
**Patients' clinical characteristics**						
DCSI score (mean ± SD)	1.02 (± 1.33)	1.50 (± 1.64)	<0.001	1.02 (± 1.33)	1.01 (± 1.30)	0.148
DCSI categories (N, %)						
0	14,089 (47.65%)	71,230 (35.10%)	<0.001	14,041 (47.57%)	14,110 (47.80%)	0.659
1	7,328 (24.79%)	50,214 (24.74%)		7,328 (24.83%)	7,358 (24.93%)	
> = 2	8,149 (27.56%)	81,500 (40.16%)		8,149 (27.61%)	8,050 (27.27%)	
CIC counts (mean ± SD)	1.21 (± 1.02)	1.26 (± 1.04)	<0.001	1.21 (± 1.02)	1.20 (± 1.01)	0.232
CIC categories (N, %)						
0	8,348 (28.24%)	54,425 (26.82%)	<0.001	8,315 (28.17%)	8,341 (28.26%)	0.765
1	10,498 (35.51%)	71,520 (35.24%)		10,483 (35.51%)	10,517 (35.63%)	
> = 2	10,720 (36.26%)	76,999 (37.94%)		10,720 (36.32%)	10,660 (36.11%)	
**Health care institution characteristics**						
Accreditation level (N,%)						
Medical Center	5,194 (16.16%)	76,958 (27.93%)	<0.001	5,194 (16.16%)	5,176 (16.11%)	0.119
Regional Hospital	11,114 (34.58%)	73,187 (26.56%)		11,114 (34.59%)	11,297 (35.16%)	
Local Hospital	6,443 (20.05%)	44,804 (16.26%)		6,441 (20.04%)	6,337 (19.72%)	
Clinics	9,385 (29.20%)	80,415 (29.18%)		9,385 (29.21%)	9,317 (28.99%)	
Ownership type (N, %)						
Public	7,079 (22.03%)	65,932 (23.92%)	<0.001	7,079 (22.03%)	6,991 (21.76%)	0.654
Not-for-profit	12,869 (40.05%)	101,656 (36.89%)		12,867 (40.04%)	12,888 (40.11%)	
For-profit	12,188 (37.93%)	107,991 (39.19%)		12,188 (37.93%)	12,255 (38.14%)	
Location of health care institution (N, %)						
Taipei	11,236 (34.96%)	90,468 (32.83%)	<0.001	11,236 (34.97%)	11,215 (34.90%)	0.828
Northern	2,924 (9.10%)	36,998 (13.43%)		2,924 (9.10%)	2,953 (9.19%)	
Central	7,653 (23.81%)	43,596 (15.82%)		7,651 (23.81%)	7,576 (23.58%)	
Southern	4,188 (13.03%)	44,657 (16.20%)		4,188 (13.03%)	4,264 (13.27%)	
Kao-Ping	4,846 (16.39%)	58,425 (28.79%)		4,846 (16.42%)	4,852 (16.44%)	
Eastern	10,403 (35.19%)	56,123 (27.65%)		10,396 (35.22%)	10,618 (35.97%)	

Note: DMHH = diabetes patients with hypertension and hyperlipidemia; DCSI = diabetes complications severity index; CIC = chronic illness with complexity; DMHH = patients with diabetes, hypertension and hyperlipidemia.

P-value here was to compare patient demographic, clinical characteristics and health institution characteristics between P4P and non-P4P patients.

Tables 3 and 4 report the incremental estimates and ICERs by direct medical costs and QALYs for P4P and non-P4P patients in both cohorts. Table 3, which compares program effectiveness, intervention costs and cost savings for the two cohort groups, shows that both P4P groups received significantly more effective care (LYs and QALYs gains) than their corresponding non-P4P groups and their cost savings were significantly greater in both cohorts (both p<0.001). Specifically, with regard to effectiveness of care, LYs for P4P and non-P4P patients were 3.93 and 3.87 in the DM alone cohort. After multiplying utility weight by LYs, those values, reinterpreted as QALYs, were 2.80 and 2.71, respectively. Adjusted incremental values per LYs gained was 0.057 and per QALYs gained was 0.082 (p<0.001) (Table 3). With regard to costs of intervention for the DM alone cohort, the difference in adjusted incremental DM-OPD costs between P4P and non-P4P patients was TWD$13,682 (USD$456) and cost savings, analyzed by adjusted incremental DM-ED/INP cost, was TWD$-14,064 (USD$-468), and by adjusted incremental all-cause medical cost, was TWD$-35,571 (USD$-1,185). S3 and S4 Tables provide full models for the analyses used to obtain Table 3 results.

**Table 3:** Incremental effects of medical costs and quality of life adjusted life years in the DM alone and DMHH cohorts.

Measures	DM alone (N = 14,267)				DMHH(N = 29,518)			
	P4P	Non-P4P	Unadjusted Increments^†^ ^‡^	Adjusted Increments^†^ ^‡^	P4P	Non-P4P	Unadjusted increments^†^ ^‡^	Adjusted increments^†^ ^‡^
	Mean ± SD	Mean ± SD	(bootstrap SE)	(bootstrap SE)	Mean ± SD	Mean ± SD	(bootstrap SE)	(bootstrap SE)
**Effectiveness**								
Life-years	3.93	3.87	0.058***	0.057***	3.86	3.80	0.063***	0.064***
	(± 0.43)	(± 0.58)	(0.006)	(0.006)	(± 0.58)	(± 0.71)	(0.005)	(0.005)
QALYs	2.80	2.71	0.083***	0.082***	2.74	2.66	0.084***	0.085***
	(± 0.31)	(± 0.42)	(0.005)	(0.004)	(± 0.42)	(± 0.50)	(0.004)	(0.004)
**Proxy of Intervention costs** ^||^								
DM-OPD costs	58,898	45,219	13,679***	13,682***	102,479	90,246	12,233***	12,314***
	(± 86,454)	(± 68,156)	(1,007)	(1,004)	(± 96,553)	(± 124,545)	(1,020)	(1,012)
**Proxy of cost-savings** ^||^ ^**¶**^								
DM-ED/INP costs	95,656	96,707	-14,730***	-14,064***	179,136	176,647	-9,744***	-9,738***
	(± 166,750)	(± 197,867)	(1,866)	(1,896)	(± 232,130)	(± 271,322)	(1,838)	(1,856)
All-cause medical costs	169,106	192,339	-36,912***	-35,571***	328,168	358,942	-43,007***	-42,850***
	(± 259,015)	(± 388,932)	(3,551)	(3,466)	(± 442,298)	(± 540,876)	(4,190)	(4,217)

Note: LYs = Life-years; QALYs = Quality adjusted life years; DM-OPD costs = Diabetes-related outpatient department costs; DM-ED/INP costs = Diabetes-related medical costs; DM alone = patients with diabetes alone; DMHH = patients with diabetes, hypertension and hyperlipidemia.

*: p<0.05

**: p<0.01

***: p<0.001

†: Incremental value here presented the value of P4P minus non-P4P with-in groups.

‡: Bootstrapped standard errors were obtained from predicted difference values from the multiple generalized linear regression models. 100 times replications with sample size equivalent to the original. Covariates that were controlled were listed in the Tables 1 and 2. Please see the supplementary S3 and S4 Tables for full models. Bootstrapped standard errors were in the parentheses.

^||^: Costs were adjusted in 2007 price based on the Taiwan National Health Insurance (NHI) global budgeting annual negotiation rate (approximately 3% discount rate). Costs are presented in Taiwan Dollar (TWD). The exchange rate between TWD and USD dollars is about 1:30 in this study.

^¶^: DM-OPD costs were not included when calculating the DM-ED/INP costs and all-cause total costs.

**Table 4:** The incremental cost-effectiveness ratio (ICER) in the DM alone and DMHH cohorts.

ICER Measures	DM alone		DMHH	
	Unadjusted increments^†^ ^§^	Adjusted increments^†^ ^§^	Unadjusted increments^†^ ^§^	Adjusted increments^†^ ^§^
	(bootstrap SE)	(bootstrap SE)	(bootstrap SE)	(bootstrap SE)
**Proxy of Intervention costs** ^||^				
DM-OPD costs per LYs gains	235,709***	240,529***	192,804***	191,968***
	(29,453)	(29,189)	(20,113)	(19,705)
DM-OPD costs per QALY gains	165,532***	167,251***	145,288***	145,474***
	(14,467)	(14,093)	(12,094)	(12,000)
**Proxy of cost-savings** ^||^ ^¶^				
DM-ED/INP costs per LYs gains	-253,819***	-247,234***	-153,577***	-151,812***
	(37,733)	(38,046)	(31,754)	(31,755)
DM-ED/INP costs per QALY gains	-178,250***	-171,914***	-115,728***	-115,043***
	(22,818)	(23,237)	(22,318)	(22,427)
All cause medical costs per LYs gains	-636,057***	-625,321***	-677,812***	-667,983***
	(86,652)	(86,431)	(92,138)	(90,343)
All cause medical costs per QALY gains	-446,684***	-434,815***	-510,767***	-506,199***
	(46,491)	(46,397)	(56,269)	(55,686)

Note: LYs = Life-years; QALYs = Quality adjusted life years; DM-OPD costs = Diabetes-related outpatient department costs; DM-ED/INP costs = Diabetes-related medical costs; DM alone = patients with diabetes alone; DMHH = patients with diabetes, hypertension and hyperlipidemia

*: p<0.05

**: p<0.01

***: p<0.001

†: Incremental value here presented the value of P4P minus non-P4P with-in groups.

§: Bootstrapping standard errors were obtained from predicted difference values from the multiple generalized linear regression models. 100 times replications with sample size equivalent to the original. Covariates that were controlled were listed in the Tables 1 and 2. Please see the S3 and S4 Tables for full models. Bootstrapped standard errors were in the parentheses.

^||^: Costs were adjusted in 2007 price using the Taiwan National Health Insurance (NHI) global budget annual negotiation rate (approximately 3% discount rate). Costs are presented in Taiwan Dollar (TWD). The exchange rate between TWD and USD dollars is about 1:30 in this study.

^¶^: DM-OPD costs were not included when calculating the DM-ED/INP costs and all-cause total costs.

**Fig 1:**
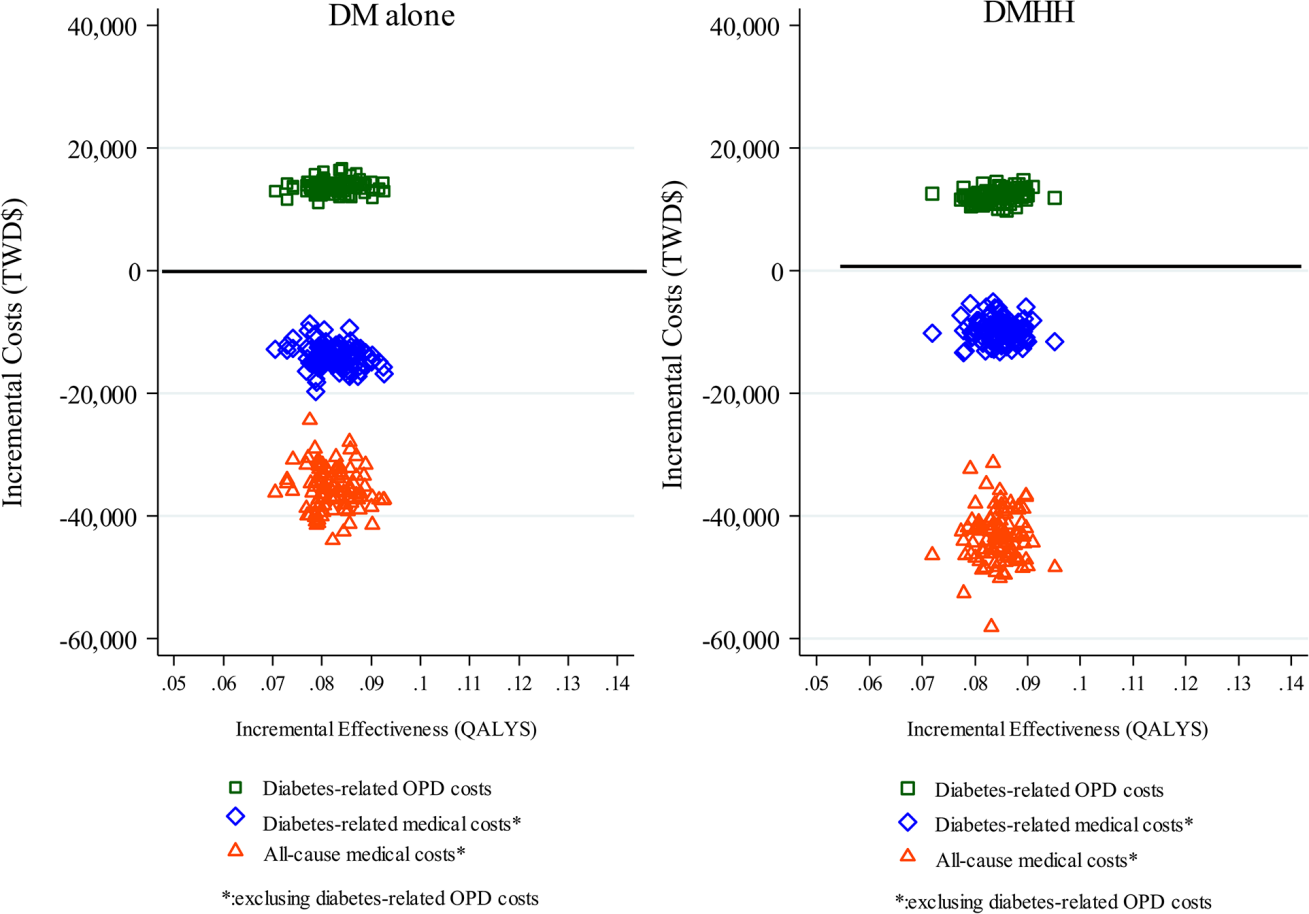
Cost-effectiveness planes for DM-OPD costs, DM-ED/INP costs and all-cause medical costs for patients with diabetes alone (DM alone) and diabetes patients with comorbid hypertension and hyperlipidemia (DMHH).

Table 4, which further analyzes Table 3 data, shows that, compared to those for non-P4P patients, the ICER for cost savings (DM-ED/INP) as well as all cause medical costs by gains in LYs and QALYs were significantly greater for P4P patients in both cohorts (all <0.001). The ICER of intervention costs (DM-OPD) for P4P patients was TWD$167,251 (USD$5,575) per QALY gained compared to non-P4P patients in the DM alone cohort, whereas the ICER of cost savings (ED-ED/INP) was for TWD$-171,914 (USD$-5,730) per QALY gained and the ICER of all cause medical costs was TWD$-434,815 (USD$-14,493) per QALY gained. In the DMHH cohort, ICER of intervention costs (DM-OPD) for P4P patients was TWD$145,474 (USD$4,849) per QALY gained compared to non-P4P patients, whereas the ICER of cost savings (ED-ED/INP) was for TWD$-115,043 (USD$-3,835) per QALY gained and the ICER of all cause medical costs was TWD$-506,199 (USD$-16,873) per QALY gained. Fig 1 shows scatter plots for the distribution of incremental QALYs and incremental costs on the cost-effectiveness planes. That figure shows cost-savings in all cause medical costs were more than twice of intervention costs in both cohorts. It also shows that the DMHH cohort had slightly lower incremental intervention costs but greater cost-savings in all cause medical costs than the DM alone cohort.

## Discussion

Although the pay for performance (P4P) has been used improve healthcare delivery and quality for patients with chronic illness, evidence of its efficiency on different level of complexity is still lacking [16, 19, 38]. In this study, we evaluated the cost-effectiveness of a P4P program for diabetes quality of care among patients with diabetes alone and with diabetes, hypertension and hyperlipidemia in Taiwan. Rather than relying on simulation modeling of the schemes’ consequences, we have directly estimated the incremental effects of costs and cost-effectiveness. For both cohorts we studied over 4-year long-term periods, we observed that the P4Ps significantly increased adjusted LYs and QALYs and made possible the cost-effective use of resources for diabetes patients.

Specifically, we found that patients enrolled in the P4P program had greater program intervention DM-OPD costs but greater cost-savings in DM-ED/INP costs as well as all-cause medical costs in both cohorts. Previous studies have found that, after program enrollment, P4P patients had significantly more diabetes-related outpatient visits, greater expense, and higher utilization of guideline-recommended services than non-P4P patients [12, 23, 25, 41]. One study also found improving trends in P4P patients’ intermediate outcomes [42]. Under such conditions, it can be expected that risks of emergency visit and hospitalizations caused by any diabetes-related complications or conditions as well as health expenses would decrease [15, 23, 25].

In addition, based on results in Table 3, the average incremental DM-OPD costs per QALYs gained was TWD$167,251 (USD$5,575) in the DM alone cohort and TWD$145,474 (USD$4,850) in the DMHH cohort, indicating the NHI had invested on the patients the P4P program over a 4-year period, and in turn, the program saved average incremental all-cause medical costs per QALYs gained of TWD$434,815 (USD$14,494) in the DM alone cohort and $506,199 (USD$16,873) in the DMHH cohort. The P4P programs were found to be cost-effective, providing a return on investment (ROI) of about 2.60:1 in the DM alone cohort and 3.48:1 in the DMHH cohorts. Similar results were reported by Curtin et al. (2006) in a study comparing overall costs of implementing and maintaining a P4P program for a health maintenance organization (HMO)’s population. Their study found the positive ROI to range 1.6 and 2.5 [43]. Hsieh et al. found the average ROI for overall diabetes patients in 2007–2011 time horizons was about 2.0[24]. These results led us to conclude that the P4P were worthy investments, especially for patients with multiple comorbid conditions.

To our best of knowledge, our research is the first paper which examined the cost-effectiveness between patients with diabetes alone and diabetes comorbid with hypertension and hyperlipidemia when compared to those without enrollment into the P4P program. Patients with multiple chronic conditions are the major users of health care services and became huge economic burdens for many countries [1]. The superior cost-effectiveness for the DMHH group might be explained by the fact that the diabetes P4P program in Taiwan requires participating healthcare providers to adhere to the American Diabetes Association (ADA)’s clinical practice guidelines for the standard care of patients with diabetes regardless of severity of disease. The guidelines contain treatment recommendations for diabetes patients with more than one common multi-morbid disease (e.g., hypertension, hypercholesterolemia, congestive heart failure, chronic kidney disease, cardiovascular disease, peripheral vascular disease, and benign prostatic hypertrophy) [14, 44]. As a result, almost all P4P program enrollees, including those in our DMHH group, benefited from the care with which they were provided. Through additional intensive follow-up care under the P4P program, patients with multiple chronic conditions may in turn reduce the risk of severe diabetes-related complications (e.g., acute myocardial infarction, stroke) and thus have greater cost-effective use of resources and cost-savings compared with those without enrollment in the program.

This study has several limitations. First, we only estimated direct medical costs paid by the NHI rather than including opportunity costs, indirect costs or NHI infrastructure /administrative costs, as suggested by Meacock et al.[19]. Second, the study was not a randomized trial: it is not possible to assign patients randomly to have or not to have a chronic disease. To classify patients in real world practice given the secondary administrative data we have in hand, we can only use ICD-9 CM diagnosis codes or drug usage to classify patients with diabetes alone or with other comorbid diseases. Third, we were not able to obtain utility score for each patient with different level of complexity in our large study sample from the secondary databases. Alternatively, we can only use survey results for diabetes patients from a cross-sectional survey as proxy measures for estimating utility weights and QALYs. The utility weights for diabetes patients were similar to those of previous studies [45]. But we were still not able to distinguish utility for patients with diabetes alone and with comorbid hypertension and hyperlipidemia. Forth, we included patients who newly enrolled in the diabetes P4P program as case group. Those P4P patients may not sustain retention in the program during four-year follow-up period in each cohort. Finally, the data we used were obtained from diabetes populations in Taiwan, so the results may not be generalized to other P4P programs in other countries.

In conclusion, there is an intrinsic challenge to move the current trend of management of individual diseases toward the care of patients with chronic comorbid illness to improve patient centered care by providing adequate support for their particular symptoms, needs, and priorities for care [3, 46]. Our analysis suggests the P4P diabetes care program in Taiwan provided long-term cost-effective use of resources and cost-savings from the perspective of return on investment for diabetes care, particularly for patients with multiple comorbid conditions.

## Supporting Information

S1 Fig Flow chart of inclusion and exclusion criteria for P4P and non-P4P patients with diabetes alone (DM alone) and with diabetes, hypertension and hyperlipidemia (“DMHH”). (DOCX)

S1 Table Lists of ATC codes for anti-hypertensive and anti-lipidemic drugs used in this study. (DOCX)

S2 Table Utility weights of health-related quality of life among P4P and non-P4P type 2 diabetes patients (≧18 years old) by age and gender. (DOCX)

S3 Table Generalized linear models results in patients with diabetes alone. (DOCX)

S4 Table Generalized linear models results in patients with diabetes, hypertension and hyperlipidemia (“DMHH”). (DOCX)

## References

[1] Tinetti ME, Fried TR, Boyd CM. Designing health care for the most common chronic condition—multimorbidity. JAMA. 2012;307(23):2493–4. 10.1001/jama.2012.5265 22797447 PMC4083627

[2] Boyd CM, Fortin M. Future of Multimorbidity Research: How Should Understanding of Multimorbidity Inform Health System Design? Public Health Reviews. 2010;32(2):451–74.

[3] Parekh AK, Barton MB. The challenge of multiple comorbidity for the US health care system. JAMA. 2010;303(13):1303–4. 10.1001/jama.2010.381 . 20371790

[4] Piette JD, Kerr EA. The impact of comorbid chronic conditions on diabetes care. Diabetes Care. 2006;29(3):725–31. . 16505540 10.2337/diacare.29.03.06.dc05-2078

[5] Wolff JL, Starfield B, Anderson G. Prevalence, expenditures, and complications of multiple chronic conditions in the elderly. Arch Intern Med. 2002;162(20):2269–76. . 12418941 10.1001/archinte.162.20.2269

[6] Isomaa B, Henricsson M, Almgren P, Tuomi T, Taskinen MR, Groop L. The metabolic syndrome influences the risk of chronic complications in patients with type II diabetes. Diabetologia. 2001;44(9):1148–54. 10.1007/s001250100615 . 11596670

[7] Freid VM, Bernstein AB, Bush MA. Multiple chronic conditions among adults aged 45 and over: Trends over the past 10 years NCHS data brief, no 100 Hyattsville, MD: National Center for Health Statistics Available: http://http://wwwcdcgov/nchs/data/databriefs/db100htm (Accessed 15 Oct 2014). 2012. 23101759

[8] Tseng LN, Tseng YH, Jiang YD, Chang CH, Chung CH, Lin BJ, et al Prevalence of hypertension and dyslipidemia and their associations with micro- and macrovascular diseases in patients with diabetes in Taiwan: an analysis of nationwide data for 2000–2009. J Formos Med Assoc. 2012;111(11):625–36. 10.1016/j.jfma.2012.09.010 . 23217598

[9] Isomaa B, Almgren P, Tuomi T, Forsen B, Lahti K, Nissen M, et al Cardiovascular morbidity and mortality associated with the metabolic syndrome. Diabetes Care. 2001;24(4):683–9. . 11315831 10.2337/diacare.24.4.683

[10] Lee TA, Shields AE, Vogeli C, Gibson TB, Woong-Sohn M, Marder WD, et al Mortality rate in veterans with multiple chronic conditions. J Gen Intern Med. 2007;22 Suppl 3:403–7. 10.1007/s11606-007-0277-2 18026809 PMC2219704

[11] Lehnert T, Heider D, Leicht H, Heinrich S, Corrieri S, Luppa M, et al Review: health care utilization and costs of elderly persons with multiple chronic conditions. Med Care Res Rev. 2011;68(4):387–420. 10.1177/1077558711399580 . 21813576

[12] Campbell SM, Reeves D, Kontopantelis E, Sibbald B, Roland M. Effects of pay for performance on the quality of primary care in England. N Engl J Med. 2009;361(4):368–78. 10.1056/NEJMsa0807651 19625717

[13] Greene J. An examination of pay-for-performance in general practice in Australia. Health Serv Res. 2013;48(4):1415–32. 10.1111/1475-6773.12033 . 23350933 PMC3725532

[14] Boyd CM, Darer J, Boult C, Fried LP, Boult L, Wu AW. Clinical practice guidelines and quality of care for older patients with multiple comorbid diseases: implications for pay for performance. JAMA. 2005;294(6):716–24. 10.1001/jama.294.6.716 . 16091574

[15] Holman RR, Paul SK, Bethel MA, Matthews DR, Neil HA. 10-year follow-up of intensive glucose control in type 2 diabetes. N Engl J Med. 2008;359(15):1577–89. 10.1056/NEJMoa0806470 . 18784090

[16] Emmert M, Eijkenaar F, Kemter H, Esslinger AS, Schoffski O. Economic evaluation of pay-for-performance in health care: a systematic review. Eur J Health Econ. 2012;13(6):755–67. 10.1007/s10198-011-0329-8 . 21660562

[17] Eijkenaar F, Emmert M, Scheppach M, Schoffski O. Effects of pay for performance in health care: a systematic review of systematic reviews. Health Policy. 2013;110(2–3):115–30. 10.1016/j.healthpol.2013.01.008 . 23380190

[18] Gillam SJ, Siriwardena AN, Steel N. Pay-for-performance in the United Kingdom: impact of the quality and outcomes framework: a systematic review. Ann Fam Med. 2012;10(5):461–8. 10.1370/afm.1377 22966110 PMC3438214

[19] Meacock R, Kristensen SR, Sutton M. The cost-effectiveness of using financial incentives to improve provider quality: a framework and application. Health Econ. 2014;23(1):1–13. 10.1002/hec.2978 . 23943496

[20] de Bruin SR, Baan CA, Struijs JN. Pay-for-performance in disease management: a systematic review of the literature. BMC Health Serv Res. 2011;11:272 10.1186/1472-6963-11-272 21999234 PMC3218039

[21] Petersen LA, Woodard LD, Urech T, Daw C, Sookanan S. Does pay-for-performance improve the quality of health care? Ann Intern Med. 2006;145(4):265–72. . 16908917 10.7326/0003-4819-145-4-200608150-00006

[22] Nahra TA, Reiter KL, Hirth RA, Shermer JE, Wheeler JR. Cost-effectiveness of hospital pay-for-performance incentives. Med Care Res Rev. 2006;63(1 Suppl):49S–72S. . 16688924 10.1177/1077558705283629

[23] Tan EC, Pwu RF, Chen DR, Yang MC. Is a diabetes pay-for-performance program cost-effective under the National Health Insurance in Taiwan? Qual Life Res. 2013 10.1007/s11136-013-0502-x . 23975377

[24] Hsieh HM, Tsai SL, Shin SJ, Mau LW, Chiu HC. Cost-effectiveness of diabetes pay-for-performance incentive designs. Med Care. 2015;53(2):106–15. 10.1097/MLR.0000000000000264 . 25397966

[25] Cheng SH, Lee TT, Chen CC. A longitudinal examination of a pay-for-performance program for diabetes care: evidence from a natural experiment. Med Care. 2012;50(2):109 10.1097/MLR.0b013e31822d5d36 22249920

[26] Chen CC, Tseng CH, Cheng SH. Continuity of care, medication adherence, and health care outcomes among patients with newly diagnosed type 2 diabetes: a longitudinal analysis. Med Care. 2013;51(3):231–7. 10.1097/MLR.0b013e31827da5b9 . 23269110

[27] Chen TT, Chung KP, Lin I, Lai MS. The Unintended Consequence of Diabetes Mellitus Pay‐for‐Performance (P4P) Program in Taiwan: Are Patients with More Comorbidities or More Severe Conditions Likely to Be Excluded from the P4P Program? Health Serv Res. 2011;46(1p1):47–60. 20880044 10.1111/j.1475-6773.2010.01182.x PMC3034261

[28] Pham HH, Schrag D, O'Malley AS, Wu B, Bach PB. Care patterns in Medicare and their implications for pay for performance. N Engl J Med. 2007;356(11):1130–9. 10.1056/NEJMsa063979 . 17360991

[29] Young BA, Lin E, Von Korff M, Simon G, Ciechanowski P, Ludman EJ, et al Diabetes complications severity index and risk of mortality, hospitalization, and healthcare utilization. Am J Manag Care. 2008;14(1):15–23. . 18197741 PMC3810070

[30] Deyo RA, Cherkin DC, Ciol MA. Adapting a clinical comorbidity index for use with ICD-9-CM administrative databases. J Clin Epidemiol. 1992;45(6):613–9. . 1607900 10.1016/0895-4356(92)90133-8

[31] Meduru P, Helmer D, Rajan M, Tseng CL, Pogach L, Sambamoorthi U. Chronic illness with complexity: implications for performance measurement of optimal glycemic control. J Gen Intern Med. 2007;22 Suppl 3:408–18. 10.1007/s11606-007-0310-5 18026810 PMC2150612

[32] Dehejia RH, Wahba S. Propensity score-matching methods for nonexperimental causal studies. The review of economics and statistics. 2002;84(1):151–61.

[33] Austin PC. A critical appraisal of propensity-score matching in the medical literature between 1996 and 2003. Stat Med. 2008;27(12):2037–49. 10.1002/sim.3150 . 18038446

[34] Brazier JE, Roberts J. The estimation of a preference-based measure of health from the SF-12. Med Care. 2004;42(9):851–9. . 15319610 10.1097/01.mlr.0000135827.18610.0d

[35] Pickard AS, Wang Z, Walton SM, Lee TA. Are decisions using cost-utility analyses robust to choice of SF-36/SF-12 preference-based algorithm? Health Qual Life Outcomes. 2005;3:11 10.1186/1477-7525-3-11 15748287 PMC555748

[36] Brazier J, Roberts J, Deverill M. The estimation of a preference-based measure of health from the SF-36. J Health Econ. 2002;21(2):271–92. . 11939242 10.1016/s0167-6296(01)00130-8

[37] Gold MR, Siegel JE, Russel LB, Weinstein MC. Cost-Effectiveness in Health and Medicine Oxford University Press: New York 1996.

[38] Drummond MF, O'Brien B, Stoddart GL, Torrance GW. Methods for the economic evaluation of health care programmes Oxford University Press: Oxford, United Kindom 1987.

[39] Weintraub WS, Cohen DJ. The limits of cost-effectiveness analysis. Circ Cardiovasc Qual Outcomes. 2009;2(1):55–8. 10.1161/CIRCOUTCOMES.108.812321 . 20031813

[40] Cromwell J, Smith KW. Evaluating Pay for Performace Interventions in "Pay for Performance in Health Care: Methods and Approaches", edited by Cromwell J, Trisolini M G, Pope G C, Mitchell J B, and Greenwald L M, RTI Press Publication No BK-0002-1103 Research Triangle Park, NC: RTI Press Accessed 15 May 2013 Available: http://wwwrtiorg/rtipress. 2011.

[41] Lee TT, Cheng SH, Chen CC, Lai MS. A pay-for-performance program for diabetes care in Taiwan: a preliminary assessment. Am J Manag Care. 2010;16(1):65–9. . 20148607

[42] Vaghela P, Ashworth M, Schofield P, Gulliford MC. Population intermediate outcomes of diabetes under pay-for-performance incentives in England from 2004 to 2008. Diabetes Care. 2009;32(3):427–9. 10.2337/dc08-1999 19106379 PMC2646022

[43] Curtin K, Beckman H, Pankow G, Milillo Y, Green RA. Return on investment in pay for performance: a diabetes case study. J Healthc Manag. 2006;51(6):365–74; discussion 75–6. . 17184001

[44] American Diabetes Association. Standards of Medical Care in Diabetes-2014. Diabetes care. 2014;37(1):S14–S80. 24357209 10.2337/dc14-S014

[45] Glasziou P, Alexander J, Beller E, Clarke P, Group AC. Which health-related quality of life score? A comparison of alternative utility measures in patients with Type 2 diabetes in the ADVANCE trial. Health Qual Life Outcomes. 2007;5:21 10.1186/1477-7525-5-21 17462100 PMC1950473

[46] Mangin D, Heath I, Jamoulle M. Beyond diagnosis: rising to the multimorbidity challenge. BMJ. 2012;344:e3526 10.1136/bmj.e3526 . 22695898

