# Supplementary material for: Cost-Effectiveness of a Diabetes Pay-For-Performance Program in Diabetes Patients with Multiple Chronic Conditions
Source: PLoS One. 2015 Jul 14;10(7):e0133163. doi: 10.1371/journal.pone.0133163 (PMC4501765; doi:10.1371/journal.pone.0133163)
Supplement: S1 Fig — (DOCX) [file pone.0133163.s001.docx]

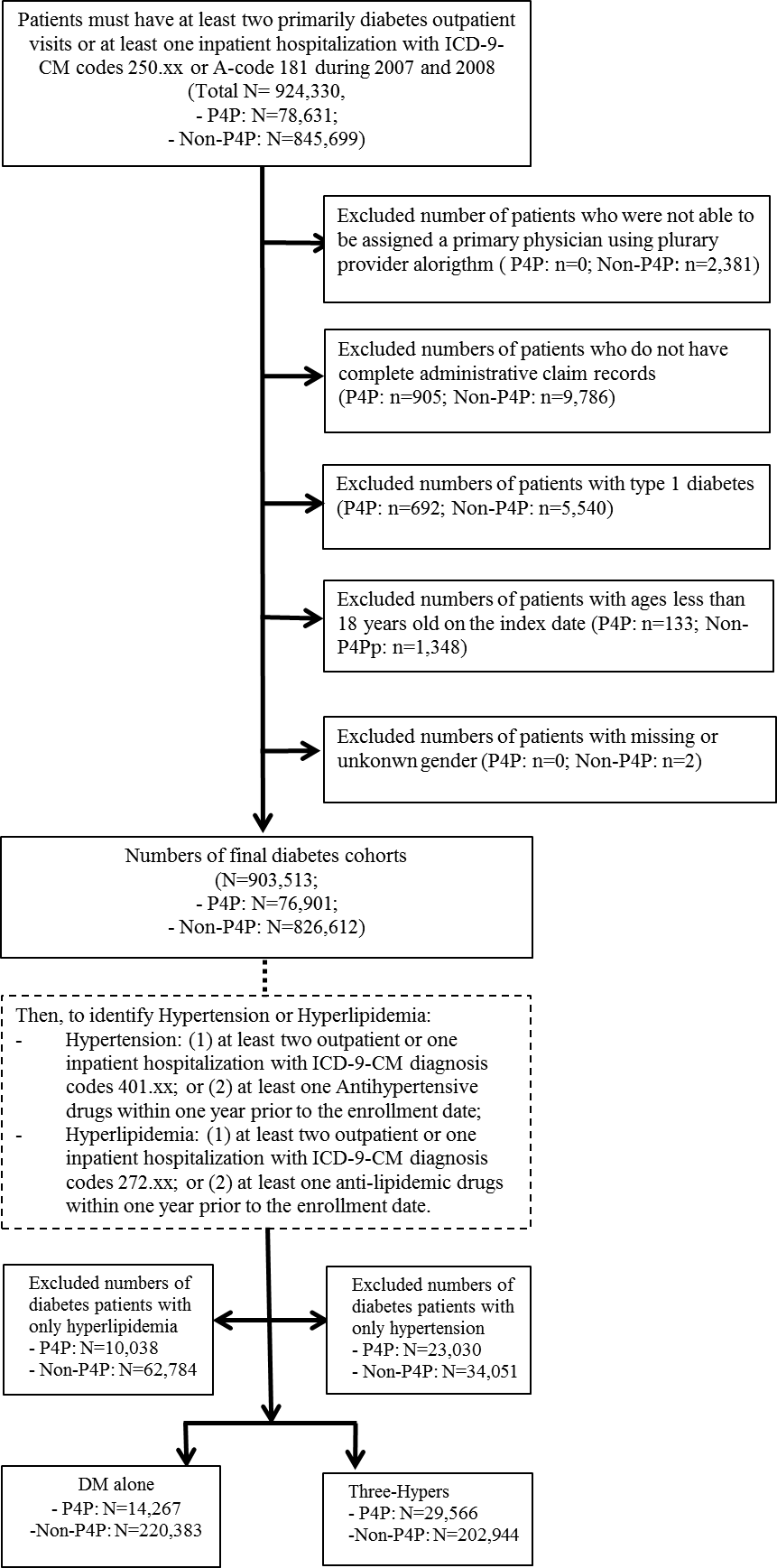


S1 Fig. Flow chart of inclusion and exclusion criteria for P4P and non-P4P patients with diabetes alone (DM alone) and with diabetes, hypertension and hyperlipidemia (“DMHH”)
