# Supplementary material for: Cost-Effectiveness of a Diabetes Pay-For-Performance Program in Diabetes Patients with Multiple Chronic Conditions
Source: PLoS One. 2015 Jul 14;10(7):e0133163. doi: 10.1371/journal.pone.0133163 (PMC4501765; doi:10.1371/journal.pone.0133163)
Supplement: S1 Table — (DOCX) [file pone.0133163.s002.docx]

S1 Table. Lists of ATC codes for anti-hypertensive and anti-lipidemic drugs used in this study

| Type | ATC_code | Drug Group |
| --- | --- | --- |
| Antihypertensive related medications | C02 | Antihypertensives |
|  | C02A | Antiadrenergic agents, centrally acting |
|  | C02C | Antiadrenergic agents, peripherally acting |
|  | C02D | Arteriolar smooth muscle |
|  | C02K | Other antihypertensives |
|  | C02L | Antihypertensives and diuretics in combination |
|  | C02N | Combinations of antihypertensives in ATC gr. C02 |
|  | C03 | Diuretics |
|  | C03A | Low-ceiling diuretics, Thiazides |
|  | C03B | Low-ceiling diuretics, excl. Thiazides |
|  | C03C | High-ceiling diuretics |
|  | C03D | Potassium-sparing agents |
|  | C03E | Diuretics and Potassium-sparing agents in combination |
|  | C07 | Beta blocking agents |
|  | C07A | Beta Blocking Agents |
|  | C07B | Beta Blocking Agents and Thiazides |
|  | C08 | Calcium channel blockers |
|  | C08C | Selective calcium channel blockers with mainly vascular effects |
|  | C08D | Selective calcium blockers with direct cardiac effects |
|  | C09 | Agents acting on the renin-angiotensin system |
|  | C09A | ACE inhibitors, plain |
|  | C09B | ACE inhibitors, combinations |
|  | C09C | Angiotensin II antagonists, plain |
|  | C09D | Angiotensin II antagonists, combinations |
|  | C09X | Other agents acting on the renin-angiotensin system |
| Antilipidemic related medications | C10 | LIPID MODIFYING AGENTS |
|  | C10A | Lipid modifying agents, plain |
|  | C10B | Lipid modifying agents, combinations |
