# Supplementary material for: Cost-Effectiveness of a Diabetes Pay-For-Performance Program in Diabetes Patients with Multiple Chronic Conditions
Source: PLoS One. 2015 Jul 14;10(7):e0133163. doi: 10.1371/journal.pone.0133163 (PMC4501765; doi:10.1371/journal.pone.0133163)
Supplement: S2 Table — (DOCX) [file pone.0133163.s003.docx]

S2 Table. Utility weights of health-related quality of life among P4P and non-P4P type 2 diabetes patients (≧18 years old) by age and gender

| Age categories | Male (N=624) | | | | | Female (N=672) | | | | |
| --- | --- | --- | --- | --- | --- | --- | --- | --- | --- | --- |
|  | P4P (N=456) | | non-P4P (N=168) | | *p-value* | P4P (N=483) | | non-P4P (N=189) | | *p-value* |
|  | N | mean ± SD | N | mean ± SD |  | N | mean ± SD | N | mean ± SD |  |
| <45 | 36 | 0.69 ± 0.15 | 19 | 0.72 ± 0.14 | 0.438 | 31 | 0.74 ± 0.15 | 15 | 0.66 ± 0.11 | 0.057 |
| 45-54 | 88 | 0.72 ± 0.14 | 27 | 0.71 ± 0.15 | 0.832 | 83 | 0.70 ± 0.15 | 28 | 0.68 ± 0.13 | 0.583 |
| 55-64 | 159 | 0.72 ± 0.16 | 63 | 0.71 ± 0.15 | 0.629 | 169 | 0.72 ± 0.17 | 68 | 0.71 ± 0.14 | 0.639 |
| 65-74 | 115 | 0.70 ± 0.16 | 37 | 0.73 ± 0.17 | 0.321 | 153 | 0.71 ± 0.16 | 47 | 0.68 ± 0.14 | 0.198 |
| 75+ | 58 | 0.70 ± 0.17 | 22 | 0.70 ± 0.18 | 0.911 | 47 | 0.69 ± 0.15 | 31 | 0.67 ± 0.12 | 0.373 |
