# Supplementary material for: Cost-Effectiveness of a Diabetes Pay-For-Performance Program in Diabetes Patients with Multiple Chronic Conditions
Source: PLoS One. 2015 Jul 14;10(7):e0133163. doi: 10.1371/journal.pone.0133163 (PMC4501765; doi:10.1371/journal.pone.0133163)
Supplement: S3 Table — (DOCX) [file pone.0133163.s004.docx]

S3 Table. Generalized linear models results in patients with diabetes alone

| **Models / Explanatory variables** | LYs^*^ | QALYs^*^ | DM-OPD costs ^*†^ | DM-ED/INP costs ^*†‡^ | All-cause medical costs^*†‡^ |
| --- | --- | --- | --- | --- | --- |
| **P4P participants** |  |  |  |  |  |
| P4P participants (Ref.=non-P4P) | 0.057*** | 0.082*** | 13,682*** | -14,064*** | -35,571*** |
|  | (0.006) | (0.004) | (916) | (1,816) | (3,640) |
| **Patients' Demographic Characteristics** |  |  |  |  |  |
| Gender |  |  |  |  |  |
| Female (Ref. group) |  |  |  |  |  |
| Male | -0.066*** | 0.002 | 563 | 13,010*** | 16,197*** |
|  | (0.006) | (0.004) | (928) | (1870) | (3708) |
| Age Categories |  |  |  |  |  |
| <45 (Ref. group) |  |  |  |  |  |
| 45-54 | -0.018** | -0.006 | 6,518*** | 4,127* | 17,035*** |
|  | (0.006) | (0.004) | (1,037) | (2,019) | (4,280) |
| 55-64 | -0.046*** | 0.017** | 6,364*** | 10,949*** | 33,297*** |
|  | (0.008) | (0.006) | (1,222) | (2,494) | (4,605) |
| 65-74 | -0.125*** | -0.075*** | 7,460*** | 28,949*** | 75,320 *** |
|  | (0.012) | (0.009) | (1,431) | (3,641) | (6,701) |
| 75+ | -0.451*** | -0.354*** | -191 | 51,292*** | 105,140*** |
|  | (0.032) | (0.022) | (1,822) | (7,727) | (12,101) |
| **Patients' Baseline Characteristics** |  |  |  |  |  |
| DCSI categories |  |  |  |  |  |
| 0 (Ref. group) |  |  |  |  |  |
| 1 | 0.003 | 0.003 | 11,035*** | 5,198 | 11,391 |
|  | (0.009) | (0.006) | (2,070) | (2,986) | (7,240) |
| >=2 | -0.108*** | -0.077*** | 20,405*** | 36,157*** | 116,176*** |
|  | (0.017) | (0.012) | (2,320) | (4,688) | (11,256) |
| CIC categories |  |  |  |  |  |
| 0 (Ref. group) |  |  |  |  |  |
| 1 | -0.010 | -0.008 | 1,508 | 4,950* | 27,200*** |
|  | (0.006) | (0.005) | (1,115) | (2,092) | (4,173) |
| >=2 | -0.051*** | -0.037*** | 2,170 | 10,658*** | 61,903*** |
|  | (0.009) | (0.006) | (1,364) | (2,450) | (4,697) |
| **Health care providers' characteristics** |  |  |  |  |  |
| Accreditation level |  |  |  |  |  |
| Medical Center (Ref. group) |  |  |  |  |  |
| Regional Hospital | -0.005 | -0.004 | -2,422 | -338 | -6,321 |
|  | (0.010) | (0.007) | (1,528) | (3,073) | (5,800) |
| Local Hospital | 0.005 | 0.003 | 396 | 2,008 | -21 |
|  | (0.011) | (0.008) | (1,877) | (3,843) | (8,831) |
| Clinics | 0.039** | 0.027** | -19,527*** | -10,013* | -18,863* |
|  | (0.012) | (0.009) | (1,801) | (4,009) | (8,145) |
| Ownership type |  |  |  |  |  |
| Public (Ref. group) |  |  |  |  |  |
| Not-for-profit | -0.015 | -0.011 | 5,294*** | 3,977 | 8,795 |
|  | (0.009) | (0.007) | (1,099) | (2,817) | (5,729) |
| For-profit | -0.018* | -0.013* | 2,566* | 1,908 | 2,379 |
|  | (0.009) | (0.006) | (1,275) | (2,514) | (4,997) |
| Location |  |  |  |  |  |
| Taipei | 0.042 | 0.030 | -7,075* | -29,832*** | -47,613*** |
|  | (0.023) | (0.017) | (2,869) | (8,513) | (13,416) |
| Northern | 0.033 | 0.023 | -12,909*** | -33,417*** | -55,260*** |
|  | (0.024) | (0.017) | (3,019) | (8,646) | (13,804) |
| Central | 0.013 | 0.009 | -3,357 | -20,952* | -31,253* |
|  | (0.024) | (0.017) | (2,981) | (8,650) | (14,284) |
| Southern | 0.018 | 0.014 | -6,036* | -17,170* | -32,320* |
|  | (0.024) | (0.017) | (3,048) | (8,722) | (14,005) |
| Kao-Ping | 0.018 | 0.012 | -6,169 | -30,647*** | -39,524** |
|  | (0.024) | (0.017) | (3,233) | (8,533) | (13,759) |
| Eastern (Ref. group) |  |  |  |  |  |
| Constant | 3.940*** | 2.720*** | 44,987*** | 64,900*** | 149,351*** |
|  | (0.027) | (0.019) | (3,562) | (9,895) | (17,432) |

Note: LYs=Life-years; QALYs=Quality adjusted life years; DM-OPD costs=Diabetes-related outpatient department costs; DM-ED/INP costs=Diabetes-related medical costs. *: p<0.05 **: p<0.01 ***: p<0.001

†: Costs were adjusted in 2007 price using the Taiwan National Health Insurance (NHI) global budget annual negotiation rate (approximately 3 % discount rate).

^‡^: Diabetes-related OPD costs were not included when calculating the diabetes related total costs and all cause total costs.
