# Supplementary material for: Cost-Effectiveness of a Diabetes Pay-For-Performance Program in Diabetes Patients with Multiple Chronic Conditions
Source: PLoS One. 2015 Jul 14;10(7):e0133163. doi: 10.1371/journal.pone.0133163 (PMC4501765; doi:10.1371/journal.pone.0133163)
Supplement: S4 Table — (DOCX) [file pone.0133163.s005.docx]

S4 Table. Generalized linear models results in patients with diabetes, hypertension and hyperlipidemia (“DMHH”)

| **Models / Explanatory variables** | LYs^*^ | QALYs^*^ | DM-OPD costs ^*†^ | DM-ED/INP costs ^*†‡^ | All-cause medical costs^*†‡^ |
| --- | --- | --- | --- | --- | --- |
| **P4P participants** |  |  |  |  |  |
| P4P participants (Ref.=non-P4P) | 0.064*** | 0.085*** | 12,314*** | -9,738*** | -42,850*** |
|  | (0.005) | (0.004) | (896) | (1,741) | (3,748) |
| **Patients' Demographic Characteristics** |  |  |  |  |  |
| Gender |  |  |  |  |  |
| Female (Ref. group) |  |  |  |  |  |
| Male | -0.062*** | 0.007 | -549 | 10,964*** | 21,579*** |
|  | (0.006) | (0.004) | (946) | (1,837) | (3,940) |
| Age Categories |  |  |  |  |  |
| <45 (Ref. group) |  |  |  |  |  |
| 45-54 | -0.015* | -0.006 | 6,101** | 617 | 10,103 |
|  | (0.007) | (0.005) | (2,085) | (3,165) | (6,982) |
| 55-64 | -0.035*** | 0.032*** | 3,797 | 1,577 | 4,568 |
|  | (0.008) | (0.006) | (1,984) | (3,215) | (6,931) |
| 65-74 | -0.141*** | -0.089*** | 389 | 25,742*** | 40,048*** |
|  | (0.009) | (0.007) | (2,042) | (3,642) | (7,438) |
| 75+ | -0.337*** | -0.279*** | -13,558*** | 41,523*** | 40,420*** |
|  | (0.014) | (0.010) | (2,177) | (4,493) | (8,801) |
| **Patients' Baseline Characteristics** |  |  |  |  |  |
| DCSI categories |  |  |  |  |  |
| 0 (Ref. group) |  |  |  |  |  |
| 1 | -0.008 | -0.005 | 10,593*** | 12,647*** | 42,015*** |
|  | (0.006) | (0.004) | (957) | (1,886) | (3,699) |
| >=2 | -0.164*** | -0.114*** | 26,987*** | 67,666*** | 230,194*** |
|  | (0.007) | (0.005) | (1,396) | (2,543) | (5,890) |
| CIC categories |  |  |  |  |  |
| 0 (Ref. group) |  |  |  |  |  |
| 1 | -0.016** | -0.012** | -1,253 | 6,543** | 22,784*** |
|  | (0.006) | (0.004) | (1,165) | (2,165) | (4,760) |
| >=2 | -0.029*** | -0.020*** | -6,124*** | 9,726*** | 50,619*** |
|  | (0.007) | (0.005) | (1,141) | (2,228) | (4,826) |
| **Health care providers' characteristics** |  |  |  |  |  |
| Accreditation level |  |  |  |  |  |
| Medical Center (Ref. group) |  |  |  |  |  |
| Regional Hospital | -0.016 | -0.011 | -2,280 | 3,956 | -6,892 |
|  | (0.008) | (0.006) | (1,648) | (2,882) | (6,296) |
| Local Hospital | -0.019 | -0.014* | -2,365 | -2,849 | -22,235** |
|  | (0.010) | (0.007) | (1,906) | (3,457) | (7,277) |
| Clinics | 0.011 | 0.007 | -36,351*** | -20,730*** | -47,038*** |
|  | (0.011) | (0.008) | (1,889) | (3,523) | (7,690) |
| Ownership type |  |  |  |  |  |
| Public (Ref. group) |  |  |  |  |  |
| Not-for-profit | -0.010 | -0.008 | 15,804*** | 4,313 | 13,667* |
|  | (0.008) | (0.005) | (1,319) | (2,623) | (5,475) |
| For-profit | -0.012 | -0.009 | 1,120 | -578 | 8,776 |
|  | (0.008) | (0.006) | (1,171) | (2,579) | (5,454) |
| Location |  |  |  |  |  |
| Taipei | 0.046** | 0.032** | -1,133 | -27,347*** | -34,379** |
|  | (0.015) | (0.011) | (2,226) | (6,241) | (11,115) |
| Northern | 0.010 | 0.007 | -10,650*** | -25,614*** | 3,022 |
|  | (0.017) | (0.012) | (2,445) | (6,584) | (12,837) |
| Central | 0.019 | 0.012 | 2,146 | -14,760* | -14,415 |
|  | (0.016) | (0.011) | (2,406) | (6,461) | (11,518) |
| Southern | 0.007 | 0.005 | -8,324** | -15,786* | -9,754 |
|  | (0.017) | (0.012) | (2,505) | (6,579) | (12,133) |
| Kao-Ping | 0.012 | 0.008 | -3,100 | -26,019*** | -25,051* |
|  | (0.016) | (0.011) | (2,451) | (6,354) | (11,584) |
| Eastern (Ref. group) |  |  |  |  |  |
| Constant | 3.948*** | 2.726*** | 90,002*** | 71,796*** | 194,544*** |
|  | (0.020) | (0.014) | (3,382) | (7,703) | (14,987) |

Note: LYs=Life-years; QALYs=Quality adjusted life years; DM-OPD costs=Diabetes-related outpatient department costs; DM-ED/INP costs=Diabetes-related medical costs. *: p<0.05 **: p<0.01 ***: p<0.001

†: Costs were adjusted in 2007 price using the Taiwan National Health Insurance (NHI) global budget annual negotiation rate (approximately 3 % discount rate).

^‡^: Diabetes-related OPD costs were not included when calculating the diabetes related total costs and all cause total costs.
